# Dynamic changes in brain network functional connectivity following repositioning treatment for benign paroxysmal positional vertigo: an fNIRS study

**DOI:** 10.3389/fneur.2026.1820148

**Published:** 2026-04-13

**Authors:** Junhua Xiao, Qingchun Pan, Renli Huang, Shu Jin

**Affiliations:** 1 Ziyang Central Hospital, Ziyang, China; 2 Affiliated Hospital of North Sichuan Medical College, Nanchong, China; 3 Longchang People's Hospital, Longchang, Sichuan, China

**Keywords:** benign paroxysmal positional vertigo, brain network, functional connectivity, near-infrared spectroscopy technology, repositioning treatment

## Abstract

**Objective:**

To explore the changes in brain network functional connectivity (FC) in patients with benign paroxysmal positional vertigo (BPPV) before and after otoconial repositioning treatment, and to analyze the correlation between FC and the Dizziness Handicap Inventory (DHI) score, thereby elucidating the neural basis of BPPV from a central mechanism perspective.

**Methods:**

A prospective cohort design was adopted, including 29 BPPV patients (BPPV group) and 29 healthy controls (control group). Resting-state brain network data were collected using 44-channel functional near-infrared spectroscopy (fNIRS), and the FC intensity of the whole brain and regions of interest (ROIs) was calculated. FC detection and DHI scoring were performed in the BPPV group before and 7 days after repositioning. Group comparisons were conducted using *t*-tests or Mann-Whitney *U* tests, and correlations were analyzed using Pearson analysis.

**Results:**

The whole-brain FC in the BPPV group before repositioning (0.52 ± 0.20) was significantly lower than that in the control group (0.64 ± 0.18) (*t* = −4.32, *P* < 0.01). Seven days after repositioning, the FC of the visual cortex V1, V2 + V3, and somatosensory cortex increased compared with that before treatment (*P* < 0.05), but did not return to normal levels (*P* < 0.05). The DHI score was negatively correlated with whole-brain FC (*r* = −0.62, *P* < 0.01).

**Conclusion:**

fNIRS revealed characteristic FC alterations in BPPV: acute-phase decreases, partial sensory cortex recovery post-repositioning, and delayed prefrontal recovery. FC correlated negatively with symptom severity, suggesting its potential as an objective biomarker. These findings provide insights into central mechanisms and support neuroregulation-assisted therapy development.

## 1 Introduction

Benign paroxysmal positional vertigo (BPPV) is the most common peripheral vestibular disorder in clinical practice, accounting for 20 to 30% of all vertigo cases. It has become a significant health issue affecting the quality of life of middle-aged and elderly people (1, 2). Patients typically experience brief vertigo and characteristic nystagmus when their head position changes. The traditional view holds that the pathogenesis is due to the dislodgement of otoliths into the semicircular canals, causing mechanical stimulation (3). However, recent studies have shown that although repositioning treatments (such as the Epley maneuver and the Barbecue roll) can eliminate nystagmus in 80 to 90% of patients, about 31 to 61% still experience residual dizziness (RD) lasting from several days to several months. This asynchronous recovery of peripheral vestibular function and symptoms suggests that the central nervous system may be involved in the disease process (4, 5).

Current research on the central mechanisms of BPPV still has significant gaps: on one hand, although fMRI and PET studies have confirmed that vestibular stimulation can activate multiple brain regions including the parieto-insular cortex, prefrontal cortex, and visual cortex, longitudinal studies on the dynamic changes of brain networks in BPPV patients are scarce (6, 7). On the other hand, conventional imaging techniques such as fMRI are limited in their application during the acute phase of vertigo, while fNIRS technology, with its strong resistance to motion interference and the ability to perform bedside monitoring, offers a new approach to exploring the interaction between the vestibular system and the cortex (8). Notably, the integration of vestibular information with visual and proprioceptive information relies on the functional coordination of distributed brain networks, and the widespread spatial orientation disorders in BPPV patients suggest that there may be reorganization of brain network connections (6).

Functional near-infrared spectroscopy (fNIRS) can non-invasively assess the functional connectivity (FC) characteristics of brain networks by detecting cortical hemodynamic responses (8). Compared with fMRI, fNIRS offers benefits such as portability, affordability, and relative tolerance to motion artifacts, making it suitable for bedside monitoring in acute vertigo patients. However, as fNIRS relies on hemodynamic responses, it is an indirect measure of neural activity and may be influenced by confounders (e.g., systemic physiological changes). Although fNIRS has been applied in vestibular disease research, studies on the dynamic evolution of brain network FC before and after repositioning treatment in BPPV patients are still lacking.

This study, based on fNIRS technology, aims to longitudinally observe the changes in whole-brain and key brain regions (prefrontal cortex, visual cortex, somatosensory cortex) FC in BPPV patients before and after repositioning treatment, and combine the Dizziness Handicap Inventory (DHI) scores to: (1) reveal the reorganization patterns of brain networks in the acute and recovery phases of BPPV; (2) clarify the association between FC changes and clinical symptoms; (3) explain the neural mechanism of residual dizziness from the perspective of central compensation. The research results will provide new evidence for understanding the central pathophysiological process of BPPV and lay the foundation for developing neuroregulation-assisted treatment plans.

## 2 Materials and methods

### 2.1 Research subjects

This study adopted a prospective cohort design and recruited subjects consecutively from the vertigo center of our hospital from January 2023 to July 2023. The sample size was calculated based on the pre-experiment data using G^*^Power software (version 3.1), with an effect size d = 0.8, α = 0.05, and β = 0.2, indicating that at least 27 cases were needed in each group. Ultimately, 58 cases were included to account for potential dropouts. The case group consisted of 29 patients with BPPV who were diagnosed for the first time and had not received repositioning treatment (17 females and 12 males). The inclusion criteria strictly followed the National “Diagnosis and Treatment Guidelines for Benign Paroxysmal Positional Vertigo” (2017 Edition) (9): Confirmed by Dix-Hallpike or supine-roll test, presenting typical positional nystagmus (such as vertical torsional nystagmus); Age 18–40 years old, excluding the interference of age-related brain function decline; Right-handed (confirmed by the Edinburgh Handedness Questionnaire); Education years >6 years, ensuring understanding of the experimental instructions; Signed informed consent, and the study was approved by the hospital ethics committee (approval number: NSMC-2022-036); Induced vertigo by head position changes, and nystagmus disappeared after repositioning treatment. The exclusion criteria included (10): History of head trauma or surgery (confirmed by CT/MRI); Secondary BPPV (such as BPPV secondary to vestibular neuritis or Meniere's disease); Central nervous system diseases (such as stroke, demyelinating diseases); Metabolic diseases (diabetes, thyroid dysfunction) or long-term use of psychotropic drugs; Mental disorders (screened by depression and anxiety scales) or cognitive impairment (MMSE score < 24).

There were 29 cases in the normal control group (13 females and 16 males). The inclusion criteria were: healthy volunteers from the community, without neurological diseases or personal history, and no previous history of vertigo attacks. Volunteers with a history of head trauma, ear diseases, other vestibular nerve lesions, neurological and mental disorders, cervical spine restrictions, inability to understand oral instructions, severe visual impairment, hearing loss, or alcohol consumption within 24 h before the assessment were excluded.

### 2.2 Testing instruments and scales

Brain function data collection was conducted using a 44-channel functional near-infrared spectroscopy imaging system (NirSmart, China). The technical parameters are as follows: the light source is dual-wavelength (730 nm, 850 nm) near-infrared laser, the detector sensitivity is >70 dB, the sampling rate is 10 Hz, and the spatial resolution is approximately 2 cm. The device calculates the activation degree of brain regions by measuring the light absorption changes of oxygenated hemoglobin (HbO) and deoxygenated hemoglobin (HbR) in cortical blood flow. BPPV diagnosis relied on the Eyeseecam vestibular function detection system (Denmark, version 2.0), equipped with a high-speed infrared camera (200 Hz) to record nystagmus trajectories; treatment was performed using an automatic repositioning device (BPPV-100, Japan), which supports standardized techniques such as Epley and Barbecue (11).

Dizziness assessment was conducted using the Dizziness Handicap Inventory (DHI) (12), this scale consists of 25 items (covering three dimensions: function, emotion, and physical), with a total score ranging from 0 to 100. The Cronbach's α is 0.92 and the test-retest reliability is 0.89. The scoring was independently completed by two blinded rehabilitation physicians. When the difference was greater than 5 points, a third physician was called in to arbitrate. The experimental environment was controlled in a soundproof room with a temperature of 22 ± 1 °C and a humidity of 50% ± 5%.

### 2.3 fNIRS resting-state data acquisition

The 44-channel portable functional near-infrared spectroscopy imaging device was used to collect resting-state brain networks, and the functional activities of the brain were studied by measuring the light scattering and absorption in the brain tissue (13).

During the test, after the subjects enter the assessment room, they first sit down comfortably and have a 5-min adaptation period to get familiar with the environment and reduce tension. Then, the subjects put on a cap and are asked to close their eyes, keeping their brains relaxed and avoiding external disturbances such as body movements. The lights in the assessment room are turned off, and the static data of the subjects are collected for 8 min (14).

**Figure 1:**
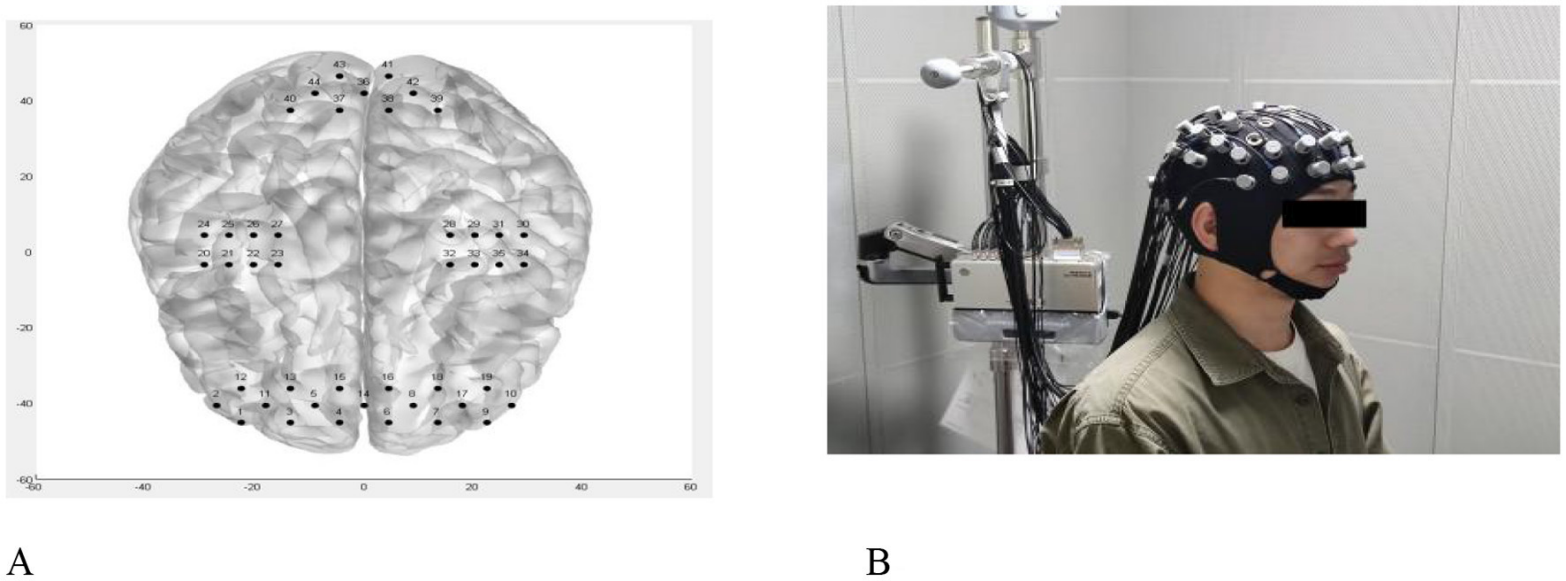
**A–B** Schematic diagram of near-infrared spectroscopy channel and head cap. **(A)** Schematic diagram of the fNIRS test area. **(B)** A. Schematic diagram of the fNIRS test area.

Based on the coverage of the probe of this device and the research objective, we divided the whole brain into five regions of interest (ROIs), namely: the prefrontal cortex, the somatosensory cortex, the motor cortex, the primary visual cortex V1, and the secondary visual cortex V2 + V3. We conducted an analysis of the functional connectivity strength between homologous and heterologous brain networks in these five ROIs. The schematic diagram of the fNIRS test area and the head cap channel are shown in Figures 1A, B.

### 2.4 Diagnosis and treatment methods of BPPV

The diagnosis of BPPV follows the standards of the International Barany Society (5): Medical history collection to confirm the characteristics of positional vertigo; Vestibular function tests: Dix-Hallpike test (posterior semicircular canal) with the head suspended at 30 °, observing the direction and latency of nystagmus; supine-roll test (horizontal semicircular canal) to record horizontal nystagmus; Cold and hot air tests (30 °C cold air, 44 °C warm air) to assess the vestibulo-ocular reflex and rule out central lesions. All tests were performed by the same experienced ENT physician.

Personalized selection of repositioning treatment: For posterior semicircular canal BPPV, the Epley maneuver (head turned 45 ° → hanging → lying on the side 180 °) is adopted; for horizontal semicircular canal, the Barbecue roll (continuous 270 ° rotation) is used; and for anterior semicircular canal, the Yacovino method (quickly sitting up—lying down) is applicable. Each technique is repeated until nystagmus disappears. A re-examination is conducted 24 h after treatment, with a success rate of over 95%. Dizziness assessment was conducted by a blinded physician using the DHI score at two time points: (1) before repositioning, when patients had active symptoms (e.g., vertigo and nystagmus) and were treatment-naive (i.e., no prior repositioning therapy); and (2) 7 days after treatment, to track short-term recovery. The assessment environment was kept consistent to minimize interference (15).

### 2.5 Data analysis and statistics

**Figure 2:**
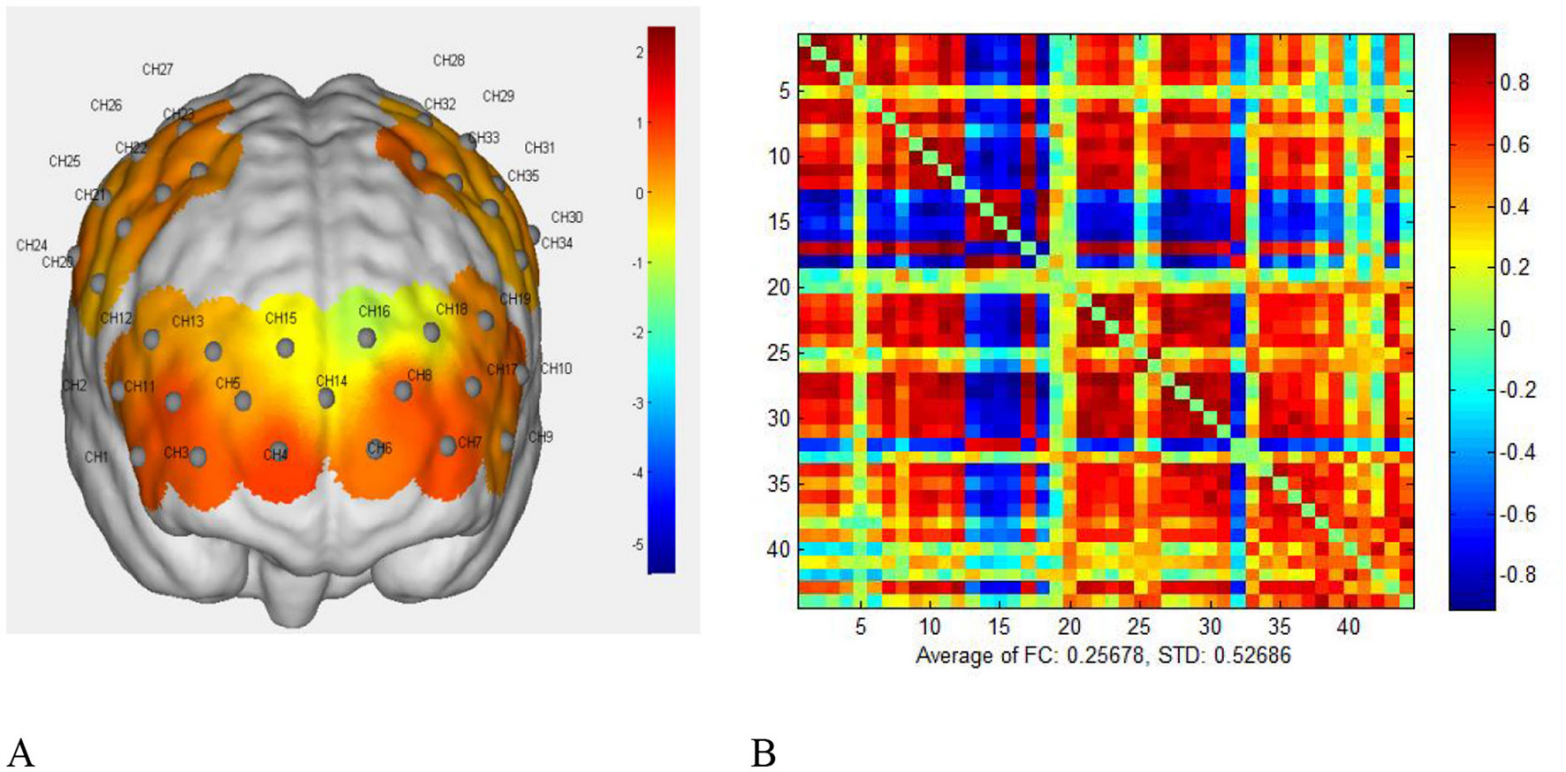
**A–B** matrix diagram of oxygenated hemoglobin and functional connectivity strength. **(A)** schematic diagram of the conversion of oxygenated hemoglobin. **(B)** Schematic diagram of the B functional connectivity strength matrix.

The fNIRS resting-state data was preprocessed using the NirSpark Preprocess module to calculate the concentration changes of the subject's oxygenated hemoglobin (HbO) and deoxygenated hemoglobin (HbR) during the resting state. In the Network module of the NirSpark software, the HbO data of the subject at each time point during the resting-state measurement was first extracted and subjected to Fisher Z transformation to convert the HbO data at different time points of the subject into a measure of functional connectivity strength (16).To minimize potential confounders in fNIRS data, such as systemic physiological noise (e.g., heart rate or respiration effects), we applied a band-pass filter (0.01–0.1 Hz) during preprocessing to isolate neural-related hemodynamic responses. This step helps reduce the influence of non-neural factors on FC estimate. See Figure 2.

Statistical analysis was conducted using SPSS 26.0 software. Measurement data conforming to a normal distribution were expressed as x ± s, and the comparison between groups was performed using the independent sample *t*-test (14); measurement data not conforming to a normal distribution were expressed as [M(P25, P75)], and the comparison between groups was conducted using the Mann-Whitney *U* test; rank data were expressed as frequency, and the comparison between groups was performed using the *U* test (15); count data were expressed as [*n* (%)], and the comparison between groups was conducted using the χ^2^ test. A *P* value < 0.05 was considered statistically significant.

## 3 Results

### 3.1 General information

There were 29 cases in the BPPV group (13 males and 16 females), with an average age of (44.97 ± 7.76). There were 29 cases in the control group (14 males and 15 females), with an average age of (42.93 ± 8.95). There were no statistically significant differences in gender, age, and educational level between the two groups (*P* > 0.05), as shown in Table 1.

**Table 1:** Comparison of general information between the Two Groups [*n* (%), ( *x* ± *s*)].

Group	number of cases	Gender (male/female)	Age (years, x ± *s*)	*Years of education (years, x ± s)*
Control group	29	14/15	42.93 ± 8.95	12.59 ± 3.75
BPPV group	29	13/16	44.97 ± 7.76	12.79 ± 3.65
χ^2^/*t* value	–	0.069	−0.925	−0.210
*P* value	–	0.792	0.359	0.834

### 3.2 Group comparisons of FC in the whole brain and ROI

**Figure 3:**
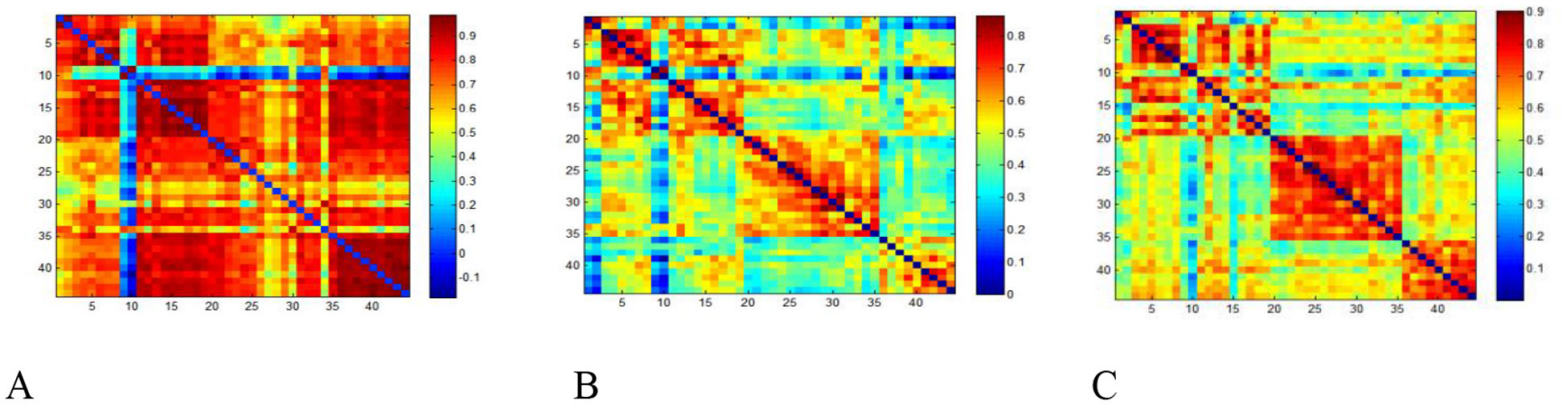
FC matrix diagram [the coordinate axes represent regions, and the correlation coefficient of each channel is set to zero (the diagonal)]. **(A)** control group. **(B)** group–Before reduction treatment. **(C)** BPPVgrou–7 days after reduction treatment.

Pearson correlation coefficients were calculated through the FC analysis of the whole brain and ROI of the cerebral cortex to determine the FC between each pair of measurement channels, generating a 44 × 44 correlation matrix for each participant. The whole brain FC intensity of the normal control group and the BPPV patients before and 7 days after repositioning treatment was analyzed. The whole brain FC intensity of the BPPV group before repositioning (0.52 ± 0.20) was significantly lower than that of the control group (0.64 ± 0.18) (*t* = −4.32, *P* < 0.01). Seven days after repositioning, the FC rose to 0.56 ± 0.14, still lower than that of the control group (*P* < 0.05). The FC matrix showed that the connection strength was concentrated in the control group, while it was dispersed in the BPPV group before treatment and partially recovered after treatment (Figures 3A, C, Table 2).

**Table 2:** Comparison of FC intensity in whole brain and ROI (x ± s).

Encephalic region	control group (*n* = 29)	BPPV group—before reset (*n* = 29)	BPPVgroup—seven days after reset (*n* = 29)	*F* value	*P* value
Whole-brain FC	0.64 ± 0.18	0.52 ± 0.20^*^	0.56 ± 0.14^*^^#^	12.36	<0.05
ROI					
Prefrontal cortex	0.58 ± 0.15	0.45 ± 0.12^*^	0.47 ± 0.10^*^	8.91	<0.05
Visual cortex V1	0.61 ± 0.16	0.48 ± 0.11^*^	0.55 ± 0.13^#^	9.24	<0.05
Visual cortex V2 + V3	0.63 ± 0.17	0.50 ± 0.14^*^	0.58 ± 0.12^#^	10.57	<0.05
Somatosensory cortex	0.59 ± 0.14	0.46 ± 0.09^*^	0.53 ± 0.11^#^	7.83	<0.05

^*^Compared with the control group, *P* < 0.05;

^#^Compared with before treatment, *P* < 0.05.

### 3.3 The changes in FC of the region of interest (ROI) after reduction treatment

Seven days after reset, the visual cortex V1, V2 + V3, and somatosensory cortex FC were significantly improved compared with those before treatment (*P* < 0.05), while the changes in the prefrontal cortex were not significant (*P* > 0.05). For details, see Table 3.

**Table 3:** Comparison of FC changes in ROI before and after re-positioning in the BPPV group.

ROI	BPPV group—before reset (*n* = 29)	BPPVgroup—seven days after reset (*n* = 29)	*F* value	*P* value
Prefrontal cortex	0.45 ± 0.12^*^	0.47 ± 0.10^*^	1.21	0.234
Visual cortex V1	0.48 ± 0.11^*^	0.55 ± 0.13^#^	4.12	<0.05
Visual cortex V2+V3	0.50 ± 0.14^*^	0.58 ± 0.12^#^	4.56	<0.05
Somatosensory cortex	0.46 ± 0.09^*^	0.53 ± 0.11^#^	3.89	<0.05

^*^Compared with the control group, *P* < 0.05;

^#^Compared with before treatment, *P* < 0.05.

### 3.4 The correlation between DHI and FC in the BPPV group before reset treatment

**Figure 4:**
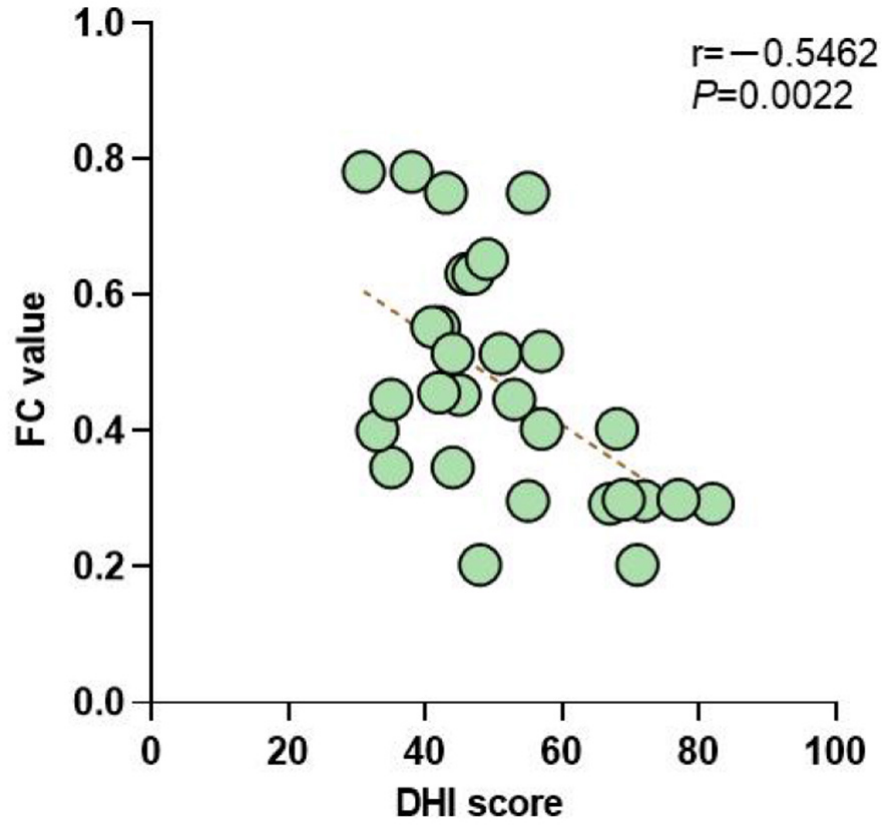
Correlation between DHI scale scores and whole-brain functional connectivity.

To investigate the correlation between the degree of vertigo and the functional connectivity of the whole brain network, we conducted a correlation study between the DHI scale score of the BPPV group and the whole brain network FC. The results showed that there was a negative correlation between the DHI scale score of the BPPV group and the whole brain network FC (*P* < 0.01), as shown in Figure 4.

**Figure 5:**
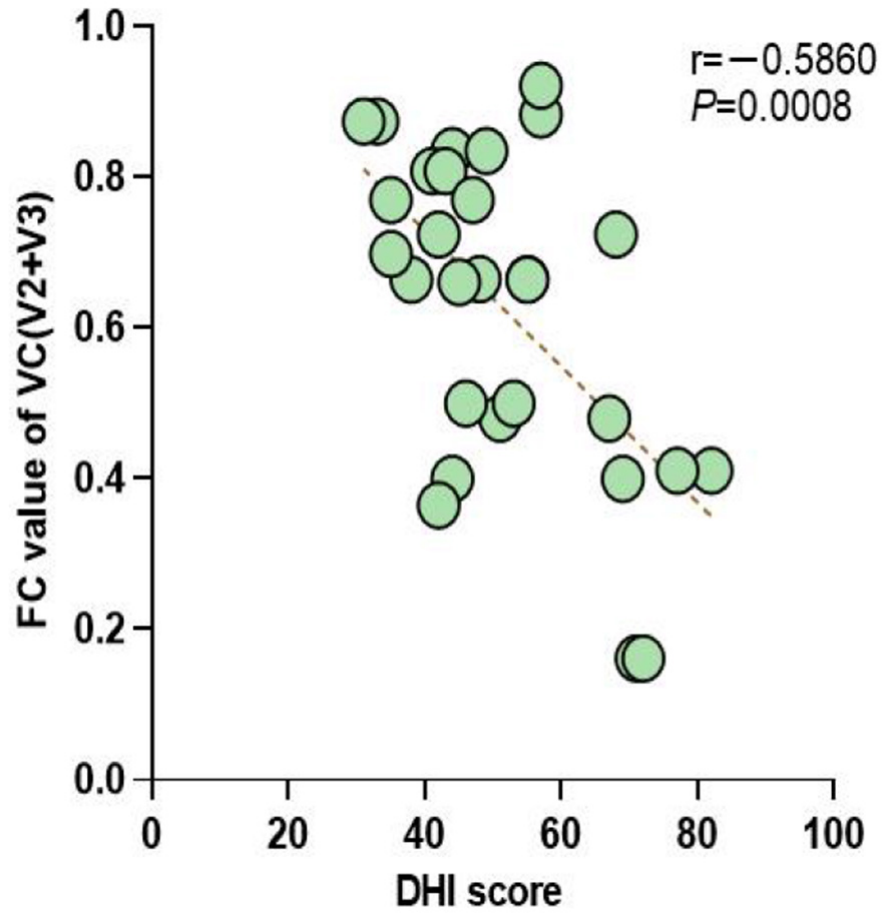
Correlation analysis between DHI scale scores and the visual cortex V2 + V3.

The first area to change in BPPV patients after repositioning treatment is the visual cortex V2 + V3. Therefore, we analyzed the correlation between the FC of the visual cortex V2 + V3 in BPPV patients and the DHI scale score. The results showed that there was a negative correlation between the DHI scale score of the BPPV group and the FC of the visual cortex V2 + V3 (*P* < 0.01), as shown in Figure 5.

## 4 Discussion

This study, systematically revealed the dynamic changes in brain network functional connectivity before and after canalith repositioning treatment in patients with BPPV through fNIRS technology. The main findings include: the whole-brain FC was significantly reduced in the acute phase of BPPV, with the somatosensory cortex and visual cortex being particularly affected; 7 days after repositioning treatment, the FC of the visual cortex and somatosensory cortex partially recovered, while the improvement in the prefrontal cortex was not obvious; the changes in FC were negatively correlated with the severity of clinical symptoms. These findings provide new evidence for understanding the central mechanism of BPPV.

This study observed that the whole-brain FC was significantly reduced in the BPPV group before repositioning. This phenomenon may reflect the cross-modal integration dysfunction caused by abnormal vestibular afferent signals. The vestibular nervous system projects widely to the parietal, temporal and prefrontal regions through the thalamocortical pathway, forming a spatial orientation functional network (6, 17).When otoconia fall off and stimulate the semicircular canal receptors, abnormal vestibular afferent signals may disrupt the balance of multisensory integration, leading to a decrease in network efficiency. FNIRS studies have shownthat vestibular stimulation in healthy individuals can synchronously activate the prefrontal-parietal network, and the decreased FC in BPPV patients in this study may reflect the impairment of this network synchrony (8). It is particularly worth noting that the FC of the sensorimotor network (somatosensory cortex) and the visual network (V1,V2 + V3 regions) decreased most significantly. This finding is consistent with the vestibular-visual-proprioceptive interaction theory (17). The vestibular, visual and proprioceptive systems jointly maintain spatial orientation function. Abnormality in any of these systems can affect the overall network function. Patients with BPPV may compensate for vestibular dysfunction by enhancing visual dependence, but this compensatory mechanism may come at the cost of network efficiency (7, 15).

Seven days after the reset treatment, we observed that the recovery of FC in the brain network showed significant regional heterogeneity and temporal dynamics. The FC in the primary visual cortex V1 area recovered from 0.48 ± 0.11 to 0.55 ± 0.13 (*P* < 0.05), that in the visual association areas V2 + V3 recovered from 0.50 ± 0.14 to 0.58 ± 0.12 (*P* < 0.05), and that in the somatosensory cortex recovered from 0.46 ± 0.09 to 0.53 ± 0.11 (*P* < 0.05). This recovery pattern conforms to the “bottom-up” neural plasticity rule: primary sensory areas recover first, while the recovery of higher-order association cortices lags behind (13, 16). The rapid recovery of FC in the visual cortex may reflect the normalization of the vestibular-visual interaction. Studies have shown that the vestibular system forms functional connections with the visual cortex through the medial superior temporal area (MST) and participates in the processing of visual flow (6, 18).After the abnormal vestibular stimulation is relieved by repositioning treatment, the visual cortex may be the first to reduce compensatory activation and restore normal network communication efficiency. The improvement of the FC in the somatosensory cortex may correspond to the process of proprioceptive recalibration, which is consistent with the clinical observation of balance function recovery in patients (15, 17). However, no significant improvement was observed in the FC of the prefrontal cortex (0.45 ± 0.12 vs. 0.47 ± 0.10, *P* > 0.05), a phenomenon with significant clinical implications. As the hub of advanced cognitive functions, the prefrontal cortex is involved in spatial working memory and attention regulation (7, 19). The delayed functional recovery of this area may explain why some patients still experience spatial disorientation and anxiety after repositioning, despite the normalization of vestibular function (4, 20).While our study focused on FC as a network-level metric, the observed decreases in visual cortex FC (e.g., V1 and V2 + V3) may reflect both local functional impairment and network disconnection. Future studies should integrate direct measures of local neural activity (e.g., EEG) to determine if compensatory enhancement occurs in the visual cortex alongside FC decreases, which would clarify the central mechanisms of BPPV.

This study found that the DHI score was negatively correlated with whole-brain FC (*r* = −0.62, *P* < 0.01), and the correlation with the FC of the visual cortex V2 + V3 area was the most significant (*r* = −0.58, *P* < 0.01). This result provides the first electrophysiological evidence for the quantitative relationship between the severity of BPPV symptoms and the degree of brain network damage. Vestibular dysfunction may affect brain network function through two pathways: one is the direct disruption of signal transmission in the vestibular-cortical pathway (6, 17); the other is the indirect triggering of negative emotions such as anxiety, affecting the function of the default mode network (4, 20). The results of this study show that the FC of sensory-related cortices (visual, somatosensory) is more correlated with symptoms than that of the prefrontal region, supporting the former as the main mechanism. This finding is consistent with the fMRI study results of Zhu et al. (17), who found that the strength of the cerebellar-parietal connection in BPPV patients was correlated with the degree of vertigo. Notably, the correlation between FC and DHI score was of moderate strength (*r* = −0.62), suggesting that clinical symptoms are also regulated by other factors, such as peripheral vestibular function status and psychological compensation ability. Future studies need to establish multivariate models to comprehensively assess the contribution of central and peripheral factors to clinical symptoms.

This study utilized fNIRS technology to overcome the limitations of traditional imaging techniques in the research of vertigo. Compared with fMRI, fNIRS has better tolerance for movement and is suitable for data collection during the acute phase (21). The 44-channel system in this study covered key nodes of the vestibular-cortical network, and its spatial resolution met the requirements for group-level analysis. Additionally, the resting-state FC analysis avoided the additional burden of task design on vertigo patients and was more in line with clinical reality (22). The longitudinal design was another major advantage of this study. By collecting data at two time points before and after repositioning, we were able to distinguish between state-dependent and trait changes in brain networks. The dynamic patterns of FC changes revealed in the results indicated that the brain network alterations related to BPPV were mainly state-dependent, providing an important reference for the time window of clinical intervention (7, 15). The findings of this study have significant implications for the clinical management of BPPV. Firstly, FC metrics may serve as objective biomarkers for evaluating therapeutic efficacy. Currently, clinical assessment mainly relies on subjective symptoms, and the quantitative indicators provided by fNIRS can assist in the judgment of therapeutic effect and prognosis prediction. Secondly, the discovery of delayed recovery of the prefrontal network offers potential targets for neuromodulation interventions (such as transcranial magnetic stimulation and transcranial direct current stimulation). For patients with significant residual dizziness, neuromodulation targeting the prefrontal cortex may promote functional recovery. Moreover, this study provided a neuroscientific basis for vestibular rehabilitation. Vestibular rehabilitation training may enhance the plasticity of the sensory integration network to promote functional compensation. Future research could combine fNIRS monitoring to personalize the adjustment of rehabilitation strategies, achieving a precise medical model of “treatment-monitoring-adjustment”.

Study limitations: Firstly, the sample size was relatively small (*n* = 29 per group), which was determined by *a priori* power analysis and sufficient for primary outcomes, but it might affect the statistical power of subgroup analyses. Future studies should involve multi-center collaborations to increase the sample size and conduct more detailed subgroup analyses, such as by different types of BPPV or stratification by disease duration, to validate the generalizability of our findings and explore potential heterogeneity. Secondly, the fNIRS technique has limited ability to monitor subcortical structures and cannot assess changes in the brainstem vestibular nuclei. Multimodal studies combining fMRI or electroencephalography may provide more comprehensive information.Thirdly, regarding the follow-up time points, only short-term changes at 7 days post-treatment were observed, and the long-term recovery process was not tracked. Future research should include additional time points such as 1 month and 3 months to depict the complete recovery trajectory. Additionally, this study did not incorporate psychological assessment indicators or assess potential comorbidities such as vestibular migraine, making it impossible to completely rule out the influence of emotional factors (e.g., anxiety, sleep quality) on FC. Subsequent work should integrate standardized scales (e.g., HADS for anxiety) to control for these confounders, particularly in patients with poor treatment response, and further explore the role of psychological factors in brain network reorganization.

## 5 Conclusion

FNIRS revealed characteristic FC alterations in BPPV: acute-phase decreases, partial sensory cortex recovery post-repositioning, and delayed prefrontal recovery. FC correlated negatively with symptom severity, suggesting its potential as an objective biomarker. These findings provide insights into central mechanisms and support neuroregulation-assisted therapy development.

## Data Availability

The original contributions presented in the study are included in the article/supplementary material, further inquiries can be directed to the corresponding author.
